# Creation of an Adiposity Index for Children Aged 6–8 Years: The Gateshead Millennium Study

**DOI:** 10.1155/2013/431825

**Published:** 2013-09-08

**Authors:** Mark S. Pearce, Peter W. James, Maria Franco-Villoria, Kathryn N. Parkinson, Angela R. Jones, Laura Basterfield, Robert F. Drewett, Charlotte M. Wright, Ashley J. Adamson

**Affiliations:** ^1^Institute of Health and Society, Newcastle University, Sir James Spence Institute, Royal Victoria Infirmary, Newcastle upon Tyne NE1 4LP, UK; ^2^Department of Statistics, Glasgow University, Glasgow G12 8QW, UK; ^3^Human Nutrition Research Centre, Newcastle University, Newcastle upon Tyne NE1 7RU, UK; ^4^Department of Psychology, Durham University, Durham DH1 3LE, UK; ^5^PEACH Unit, Faculty of Medicine, Glasgow University, Glasgow G12 8QQ, UK

## Abstract

*Objective*. A number of measures of childhood adiposity are in use, but all are relatively imprecise and prone to bias. We constructed an adiposity index (AI) using a number of different measures. *Methods*. Detailed body composition data on 460 of the Gateshead Millennium Study cohort at the age of 6–8 years were analysed. The AI was calculated using factor analysis on age plus thirteen measures of adiposity and/or size. Correlations between these variables, the AI, and more traditional measures of adiposity in children were investigated. *Results*. Based on the factor loading sizes, the first component, taken to be the AI, consisted mainly of measures of fat-mass (the skinfold measurements, fat mass score, and waist circumference). The second comprised variables measuring frame size, while the third consisted mainly of age. The AI had a high correlation with body mass index (BMI) (rho = 0.81). *Conclusions*. While BMI is practical for assessing adiposity in children, the AI combines a wider range of data related to adiposity than BMI alone and appears both valid and valuable as a research tool for studies of childhood adiposity. Further research is necessary to investigate the utility of AI for research in other samples of children and also in adults.

## 1. Introduction

It is well known that the prevalence of childhood obesity has increased rapidly in most parts of the world [1]. While the recent evidence suggesting a levelling off in the incidence of childhood overweight and obesity is promising [2], the prevalence of excess weight in children continues to be an urgent public health challenge. Childhood obesity is known to be an important risk factor for future morbidity and risk of early mortality [3]. Obese children are also more likely to experience psychological or psychiatric problems than nonobese children, and the risk of psychological morbidity increases with age [3, 4]. While the presence of high rates of childhood obesity at population levels is evident, it is less clear how to best identify children with excess adiposity at an individual level. There are a number of measures of childhood adiposity currently in use, all of which are relatively imprecise and prone to bias.

By definition, obesity is excess body fatness and should ideally be defined on the basis of a measure of body fatness. However, all gold standard methods of measurement are expensive or invasive so that simpler proxy measures of excess fatness are usually required [5]. Body mass index (BMI) (weight (kg)/height (m)^2^) is widely accepted as a convenient measure of a person's fatness, but it does not separate fat from lean mass and thus provides a screening but not a diagnostic test [6]. This is a particular issue in children where BMI alone cannot accurately distinguish healthy, muscular children from those who are obese. Waist circumference is a measure of abdominal fat and has proven useful for assessing obesity-related risks of diseases [7]. Skinfold measurements measure subcutaneous fat, and measuring triceps and subscapular skinfold thicknesses has been recommended as part of medical assessments for children and adolescents [8, 9]. Bioelectrical impedance measures total lean mass from which fat mass is inferred by subtraction. It is widely used in adults but less so in children for whom the standard childhood conversion formulae relate poorly to gold standard methods [10, 11]. However, recent work has produced norms for fat and lean mass in mid-childhood [12]. Dual-energy X-ray absorptiometry (DXA) is also widely used to assess fat mass but is not suitable for field or clinic work, and its estimates can also agree poorly with the gold standard in childhood [11]. However, none of the measures correspond closely to gold standard methods, and none are obviously superior to the others [11].

When we set out to assess adiposity in children from the Gateshead Millennium Study (GMS), recognising that no one measure is reliable in terms of assessing childhood adiposity, we collected a number of different measures. However, in order to avoid the use of multiple endpoints and significance tests, we aimed from the outset to combine these into a single “adiposity index” (AI), which allows the addition of other aspects of adiposity measurement, such as skinfolds, within the same measure, thus creating an average value that should be more precise and accurate. This paper describes how this index was constructed and how it relates to known correlates of adiposity and to the most widely used measure, BMI.

## 2. Methods

### 2.1. Participants

The GMS is a birth cohort study which initially comprised 1029 infants and their families recruited shortly after birth between June 1999 and May 2000 in Gateshead, an urban district in north east England. Full details of recruitment and measures taken since birth are reported elsewhere [13, 14]. The sample was slightly overrepresented at the lower end of the socioeconomic scale at recruitment but due to sample attrition was representative of north east England for the present study in 2006/2007 [14]. The data reported and described here were collected at the 6–8-year data sweep which had a primary focus of investigating whether modifiable predictors of childhood adiposity could be identified. A favourable ethical opinion was obtained from the Gateshead Local Research Ethics Committee for this study. Informed written consent was obtained from the parent/main carer of each child, and children provided assent to their participation.

### 2.2. Anthropometric Measurements

All equipment for this study was supplied by Chasmors (London, UK). All measurements were by project staff trained in the same way to ensure standardisation of measurements across project staff. Children were measured barefoot and in light indoor clothing. Duplicate measurements of all variables except bone frame were taken, and means were calculated and used in analysis. Height was measured to 0.1 cm with a Leicester Portable height measure, and weight was measured to 0.1 kg (Tanita TBF-300MA), from which BMI was derived. International Obesity Taskforce (IOTF) references were used to classify children into four categories (“underweight,” “healthy weight,” “overweight,” and “obese”) [15, 16]. *z*-scores were also created for BMI according to the UK90 standards [17].

Waist circumference was measured to 0.1 cm at the minimum circumference between the lowest rib and the iliac crest without compressing the skin. *z*-scores for waist circumference were created according to the UK 1988 standards [18, 19]. Skinfolds were measured to 0.1 mm on the child's nondominant side using a Holtain skinfold calliper in the following positions: biceps (midline of the anterior aspect of the arm); triceps (midline of the posterior aspect of the arm); subscapular (inferior to the inferior angle of the scapula); suprailiac (positioned approximately 1 cm above and 2 cm medial to the anterior superior iliac spine) [20–23]. Bioelectrical impedance was measured using a Tanita TBF-300MA and expressed as lean and fat mass scores adjusted for height, sex, and age [12]. Bone frame size was measured to 0.1 cm at the following sites: shoulder (biacromial) and hip (bi-iliac) were measured using a Harpenden Anthropometer, and knee (bicondylar femur), wrist (across the styloid process), and elbow (across the humeral epicondyles) were measured using a Harpenden Bicondylar Calliper [24, 25].

### 2.3. Statistical Analysis

One twin per twin pair (chosen at random) was included to ensure independence. Factor analysis, with a promax rotation, on the correlation matrix of logged data was undertaken for age plus the thirteen measures of size and/or adiposity: height, width of shoulders, diameters of elbow, wrist, hip, and knee, waist circumference, skinfold measures of the subscapular, triceps, biceps, and suprailiac, and the impedance-based measures of fat and lean masses expressed as scores. An AI was then constructed using the first component, which was dominated by the adiposity measures. The correlation of this index was explored with each individual measure included in the factor analysis, as well as with BMI. Additional comparisons were made with the IOTF categories [15, 16] and with the externally referenced *z*-scores for BMI [17] and waist circumference [18, 19]. As only height, shoulder bone frame size, and the lean mass score were normally distributed, the Spearman rank correlation was used for all pairwise correlations, and medians (and corresponding interquartile ranges (IQRs)) are reported. Differences between two groups (e.g., sex) were tested using the Wilcoxon rank sum tests, with the Kruskal-Wallis test used for tests between more than two groups. Statistical analysis was carried out using the statistical software package Stata, version 10 (StataCorp, College Station, TX, USA).

## 3. Results

Complete body composition data were available for 460 children, 227 boys and 233 girls. Descriptive data on all of the variables in the factor analysis are given in Table 1. The average age of the children included was 7.5 years, with no significant age difference between boys and girls. Significant differences between boys and girls were however seen for all four skinfolds, with higher average skinfolds in girls. Boys had higher averages for bone frame measures of the elbow and knee, while girls had higher lean mass scores (Table 1).

A three-component model was found to explain 79% of the overall variance between all the adiposity- and size-related measures included in the factor analysis. On the basis of the size of the loadings (Table 2), the first component (taken to be the AI) consisted mainly (in terms of factor loadings, all variables were included in all components) of measures of fat mass (waist circumference, the four skinfold measurements, and fat mass score). The second component consisted mainly of variables measuring frame size (height, shoulders, elbow, wrist, knee, and lean mass score), while the third consisted mainly of age. Similar factor analyses were done for boys and girls separately, with similar results found for both in terms of weightings for all components. Hence, only the combined index is presented.

The median AI overall was −0.14 (IQR −0.69, 0.50) and was lower for boys (−0.52 (IQR −0.91, 0.25)) than it was for girls (0.13 (IQR −0.43, 0.83)).

The created AI (i.e., the first component) was significantly correlated with both of the other components (*r* = 0.55 with the second component, the “frame size component,” and *r* = 0.18 with the third component, the “age component,” *P* < 0.001 for both). Correlations between the measures included in the factor analysis, BMI, and the created AI are given in Table 3. Most correlations were significant at the *P* < 0.001 level, although of varying degrees of magnitude. The AI was significantly correlated with all other variables, although with age the correlation was small (rho = 0.14, *P* value = 0.003). Correlations with the AI were greatest for the fat mass variables and BMI and lowest for the variables with the greatest weights in the second component (i.e., those related to frame size). While age was significantly correlated with the frame size measures and BMI (*P* < 0.001), it was less significantly so with the subscapular (*P* = 0.001), triceps (*P* = 0.03), and suprailiac (*P* = 0.009) skinfolds and lean mass score (*P* = 0.04) and not significant for biceps skinfold (*P* = 0.08) or fat mass score (*P* = 0.19).

According to IOTF reference values, three children (0.7%) in this study were underweight, 348 (76%) were of healthy weight, 77 (17%) were overweight, and 32 (7%) were obese. There was a significant association between the IOTF groups and the created adiposity index (*P* = 0.0001). The median AI was −0.44 (IQR −0.83, 0.03) in the healthy weight group, 0.88 (IQR 0.48, 1.23) in the overweight group, and 2.25 (IQR 1.75, 2.84) in the obese group. The AI was also highly correlated with both the *z*-scores created for BMI using the UK90 standards (rho = 0.79, *P* < 0.0001) and those for waist circumferences (rho = 0.77, *P* < 0.0001).

## 4. Discussion

The created AI was primarily constructed from variables considered *a priori* to be related to fat mass rather than frame size or lean mass. The AI created using these variables showed the expected sex variation and was highly correlated with BMI, fat mass, waist circumference, and the skinfold measures. It was also as would be expected, significantly associated with three external standards, the IOTF reference cutoffs for BMI [15, 16], *z*-scores for BMI using the UK90 standards [17], and *z*-scores for waist circumference [18, 19].

The main strength of this study is that a wide range of measures of body composition were measured in an unselected population of children. The single measure described here is a research tool which has enabled our study to explore influences on childhood adiposity using one primary outcome measure rather than suffer the problems associated with multiple testing of correlated outcome measures.

The differences in correlations between the various measures of adiposity suggest that they do not measure exactly the same thing. This is in keeping with previous studies that have found that none of the field-based measures produce precise or unbiased estimates of true fat mass [11]. However the finding that the measures group together in a factor separate from size suggests that they are all measuring elements of a child's adiposity and that the different methods used would be expected to produce better estimates of, respectively, subcutaneous (skinfolds), abdominal (waist), and total (bioelectrical impedance) body fat. The significant association of the AI with height also implies that epidemiological studies using an AI such as the one in this paper should also carefully consider whether height may play a confounding role in predicting adiposity.

However, as a tool for clinical use, the AI is limited as it would require all of the measures of body size and composition taken in this study to be taken and then a complex formula to be applied based on the factor loadings and the included variables, all of which may differ in different populations. Hence, further creations of similar adiposity indices in different age groups and different populations would be needed before formulae could be created for clinical use. Both waist and, particularly, skinfolds measurements are subject to both intra- and intermeasurer errors and require extensive anthropometrist training as was given in this study. Both measures require the removal of some clothing, which may be unacceptable in some settings. Away from a clinical setting, privacy screens are required (extra equipment to purchase and transport, increasing the area and time required for setting up on site) and extra staff to serve as chaperones. As skinfolds can be uncomfortable, children may object to them, particularly to duplicate measures being taken. Waist measures, though practical, can be problematic as many children are “ticklish” and therefore move whilst the measure is being taken or hold their breath/expand their stomach and are unable to “breathe normally” as required for the optimum measurement. Height and weight measurements require less training, are time efficient, and require only an accurate scale and stadiometer which are widely available in most clinical settings and easily transported to field settings. Importantly, height and weight measures are also generally highly acceptable to children. Bioelectrical impedance is a practical and easily obtained method. However, interpretation of the measurements made is more problematic as values reported by the machines used rely on standard equations not necessarily valid for children. Further, good bioelectrical impedance measures depend on factors such as whether the child's bladder is empty, if they are normally hydrated, which can be particularly an issue for early morning measurements, and unclean feet may interfere with conductivity.

McCarthy et al. [19] recommended that a simple measure of overweight and obesity is needed to be developed for use in populations, with ease of use for clinical and field settings. They suggested waist circumference be routinely measured. However, to allow comparison between datasets, internationally agreed sites of measurement need to be established. Differences between sexes, ethnic groups, and age of children affect distribution of fat patterning, and thus standards representing children of all backgrounds are required. A study of 8–11-year-old children in South Africa concluded that although the waist circumference index (waist (cm)/height (m)) was a useful approximation of body fat as measured by DXA scanning, BMI was a more accurate and convenient tool in prepubertal children [26]. Del Mar Biblioni et al. [27] recently reported that there is a misclassification when adiposity is considered using the IOTF and WHO-2007 international references. By adding an estimate of both adiposity, such as the fat mass index used in our study, and fat distribution, such as waist : hip ratio, they developed the AFAD-A classification and suggested that this could be useful in identifying overfat adolescents, rather than just overweight. However, their study was in adolescents (aged 12–17), so different findings may have been seen in a much younger population such as ours.

Olds and colleagues conducted a meta-analysis of young people (under 19 years old) looking at secular trends in fatness using skinfold thicknesses. They showed significant increases in skinfold thicknesses, in line with the increases in childhood obesity as measured by other techniques [28]. They report that different methodologies used to measure skinfold thicknesses increase variability (such as slightly different site locations, different types of callipers, use of right versus left side of the body, single versus multiple measurements, and different degrees of anthropometric training) but that skinfolds can be measured accurately and reliably by trained anthropometrists. What no study has ever considered for mass data collection is a composite measure of fat based on multiple measurement modalities, although this is now proposed as a gold standard method for laboratory-based body composition measures [29]. While this measure is plausible, we cannot be sure that it produces a more precise or less biased estimate of body fat until it has itself been compared to a gold standard method such as that using stable isotopes. What this study does confirm is that BMI is a simple and practical proxy measure of adiposity, where more specific measures of body composition are not required. However, creating an AI allows the addition of other aspects of adiposity measurement, such as skinfolds, within the same measure, thus creating an average value that should be more precise and accurate. While it is unlikely to be of wide use in clinical settings, it is likely to be a valuable research tool.

## 5. Conclusion

An AI developed using a combination of thirteen different measures of body size and composition is, reassuringly, best explained by measures that would be thought to measure childhood fat mass. While BMI is a highly practical tool for screening and assessment of adiposity in children of this age group, the AI combines a wider range of data related to adiposity than BMI alone, and using this methodology appears to be both valid and valuable as a research tool for studies of childhood adiposity, although; given the limitations of some of the components, its validity may vary for different childhood age groups. Further research is necessary to investigate the utility of AI for research in other samples of children and also in adults.

## Figures and Tables

**Table 1:** Descriptive statistics for the variables included in this study, by sex.

Variable	All median (IQR)	Boys median (IQR)	Girls median (IQR)	*P* for sex difference
Age (years)	7.5 (7.2, 7.8)	7.4 (7.2, 7.8)	7.6 (7.2, 7.8)	0.53
Height (cm)	125 (122, 129)	126 (122, 130)	125 (121, 128)	0.09
Shoulders (mm)	279 (269, 291)	279 (270, 291)	278 (268, 290)	0.51
Elbow (mm)	52 (49, 54)	52 (50, 55)	51 (49, 53)	0.001
Wrist (mm)	42 (41, 45)	43 (41, 45)	42 (40, 44)	0.07
Hip (mm)	203 (194, 213)	202 (194, 213)	204 (195, 214)	0.35
Knee (mm)	77 (74, 80)	78 (75, 82)	75 (73, 79)	<0.0001
Waist (cm)	55.9 (53.0, 59.3)	56.4 (53.3, 58.8)	55.4 (52.5, 59.6)	0.14
Subscapular skinfold (mm)	6.6 (5.3, 9.6)	5.8 (4.9, 8.2)	7.7 (5.9, 10.5)	<0.0001
Triceps skinfold (mm)	10.5 (8.3, 13.4)	9.0 (7.3, 11.7)	11.6 (9.7, 14.3)	<0.0001
Biceps skinfold (mm)	6.1 (4.7, 8.2)	5.5 (4.4, 7.3)	6.7 (5.4, 8.7)	<0.0001
Suprailiac skinfold (mm)	7.9 (5.5, 11.9)	6.7 (5.0, 10.1)	9.5 (6.1, 14.0)	<0.0001
Fat mass score	0.44 (0.00, 0.94)	0.54 (0.06, 1.01)	0.39 (−0.03, 0.83)	0.06
Lean mass score	−0.20 (−0.80, 0.48)	−0.30 (−0.89, 0.34)	−0.07 (−0.68, 0.56)	0.01
BMI (kg/m^2^)	16.3 (15.2, 17.8)	16.2 (15.3, 17.5)	16.5 (15.2, 18.0)	0.18

IQR: interquartile range.

BMI: body mass index.

**Table 2:** Loadings for each of the three components created in the factor analysis, for each variable included within it.

Variable	Component 1	Component 2	Component 3
Age (years)	−0.008	0.002	0.972
Height (cm)	−0.053	0.829	0.216
Shoulders (mm)	0.110	0.720	0.168
Elbow (mm)	0.104	0.774	−0.108
Wrist (mm)	0.012	0.838	−0.096
Hip (mm)	0.409	0.422	0.040
Knee (mm)	0.020	0.904	−0.099
Waist (cm)	0.597	0.426	−0.011
Subscapular skinfold (mm)	0.934	0.011	0.004
Triceps skinfold (mm)	0.963	−0.070	−0.028
Biceps skinfold (mm)	0.954	−0.041	−0.058
Suprailiac skinfold (mm)	0.899	0.047	−0.020
Fat mass score	0.907	−0.072	0.109
Lean mass score	−0.135	0.990	−0.005

**Table 3:** Correlations (rho) between the created adiposity index, all variables included in the factor analysis, and BMI.

Variable	Adiposity index	Age	Height	Shoulders	Elbow	Wrist	Hip	Knee	Waist	Subscapular skinfold	Triceps skinfold	Biceps skinfold	Suprailiac skinfold	Fat mass score	Lean mass score
Age	0.14														
Height	0.39	0.33													
Shoulders	0.51	0.29	0.77												
Elbow	0.45	0.18	0.59	0.57											
Wrist	0.40	0.18	0.62	0.58	0.60										
Hip	0.65	0.24	0.68	0.68	0.54	0.55									
Knee	0.43	0.21	0.68	0.65	0.69	0.66	0.61								
Waist	0.75	0.23	0.57	0.65	0.56	0.55	0.70	0.63							
Subscapular skinfold	0.92	0.15	0.35	0.48	0.45	0.38	0.56	0.42	0.69						
Triceps skinfold	0.92	0.10	0.30	0.40	0.41	0.34	0.52	0.40	0.61	0.83					
Biceps skinfold	0.90	0.08	0.29	0.38	0.39	0.34	0.53	0.35	0.62	0.80	0.83				
Suprailiac skinfold	0.91	0.12	0.39	0.51	0.42	0.43	0.59	0.44	0.70	0.84	0.82	0.80			
Fat mass score	0.72	0.06	0.14	0.28	0.33	0.19	0.41	0.27	0.58	0.63	0.60	0.61	0.57		
Lean mass score	0.24	0.09	0.11	0.27	0.32	0.35	0.26	0.40	0.38	0.30	0.28	0.26	0.29	−0.11	
BMI	0.81	0.17	0.41	0.56	0.58	0.51	0.64	0.62	0.83	0.77	0.73	0.72	0.74	0.64	0.58

BMI: Body Mass Index.
